# The Medical Attitude Towards Compulsory Vaccination

**Published:** 1896-08-29

**Authors:** 


### The Medical Attitude Towards Compulsory Vaccination.

The aim of medicine, as the guardian of the common health, must, at the present moment, be to secure as far as possible universal vaccination and re-vaccination. The question of practical importance is, how may this best be done? No doubt the "short way" with recalcitrants is always the simplest; and it is a method which invariably commends itself to a certain order of minds. But modern civilization, we think very properly, objects to the rack and the thumbscrew, even though they take in these days the very modified forms of fines and short terms of imprisonment.

Medicine owes something to herself in this matter. She is not, as she was at the beginning of the century, largely ignorant, and therefore excessively dogmatic. She has taken Bacon's advice to heart, and become an adept in the use of the methods of induction. And just in proportion as she has found how difficult it is to make large generalisations whose foundations can be shown to be absolutely and unassailably secure, so she has become less dogmatic year by year, and inclined rather to the methods of persuasion than to those of force. No doubt many among us still hold, with the "voluminous" Gibbon, that "persuasion is the resource of the feeble"; but a still larger number, we would fain hope, are convinced that "persecution is the method of the stupid," and a most useless weapon it usually is when it is attempted to be practised on a high-spirited and freedom-loving people.

What, then, is our resource as convinced vaccinators and re-vaccinators? Instruction! If we attempt now to insist upon drastic measures of compulsion the effect will almost certainly be to defeat the very object we have in view. Our desire is to secure that vaccination and re-vaccination shall be as widely performed as possible. Our initial methods should be, in the first place, to convince the reason of the people; and, in the second, to make vaccination as easy of administration and as safe to the subject of it as it can possibly be made.

For a time, at least, our profession as a profession, should be content, except so far as instruction may be fitly carried out, with a "masterly inactivity." The full text of the Report of the Royal Commission will soon be available for every person who is disposed to study it. The business of the medical man who has any regard for the reputation of his profession for statesmanship will be to study the facts of the report; to study them again and again; to study them from the point of view, not merely of the sanitarian, but from the larger point of view of the reasoning and practical statesman. In a country like our own, with a large population and a very small area, we are compelled to believe in "Health by Act of Parliament." We cannot help ourselves. If we do not legislate for sanitary safety we shall have no sanitary safety. But, all this notwithstanding, our aim as medical men must be to keep the end steadily in view, the great end of general vaccination and re-vaccination, and for this purpose, we insist, we should be anxious to show the public that we are as willing to take ample time for the reconsideration of the whole question as the public is. One year, five years, ten years do not constitute a long period in the life of the nation. The Commission, we believe, has brought to light facts which prove to demonstration that the old Jennerian view of "one vaccination, life-long protection" is no longer tenable. Indeed, everybody but the most stupid of persons knew that before the Commission commenced its sittings.

In short, our contention is that we have a strong position, and, therefore, that we can afford to be calm and wait. The evidence of the Commission, though it will no doubt make clear to us all that we have no mathematical certainty of the effect of vaccination in any given case, will yet show that in vaccination and re-vaccination we have prophylactics of such a high degree of value that no country can be considered "sane" which does not adopt and make full use of those prophylactics. That being undoubtedly so, if we as a profession show ourselves strong and reasonable, it may be that in no long time opposition, being not opposed, will die down, and that the democracy will of itself adopt universal vaccination and re-vaccination, even with a modified compulsion attached.

